# Surgical management of inguinal hernias at Bugando Medical Centre in northwestern Tanzania: our experiences in a resource-limited setting

**DOI:** 10.1186/1756-0500-5-585

**Published:** 2012-10-25

**Authors:** Joseph B Mabula, Phillipo L Chalya

**Affiliations:** 1 Department of Surgery, Catholic University of Health and Allied Sciences-Bugando, Mwanza, Tanzania

**Keywords:** Inguinal hernias, Surgical management, Treatment outcome, Predictors of outcome, Tanzania

## Abstract

**Background:**

Inguinal hernia repair remains the commonest operation performed by general surgeons all over the world. There is paucity of published data on surgical management of inguinal hernias in our environment. This study is intended to describe our own experiences in the surgical management of inguinal hernias and compare our results with that reported in literature.

**Methods:**

A descriptive prospective study was conducted at Bugando Medical Centre in northwestern Tanzania. Ethical approval to conduct the study was obtained from relevant authorities before the commencement of the study. Statistical data analysis was done using SPSS software version 17.0.

**Results:**

A total of 452 patients with inguinal hernias were enrolled in the study. The median age of patients was 36 years (range 3 months to 78 years). Males outnumbered females by a ratio of 36.7:1. This gender deference was statistically significant (P = 0.003). Most patients (44.7%) presented late (more than five years of onset of hernia). Inguinoscrotal hernia (66.8%) was the commonest presentation. At presentation, 208 (46.0%) patients had reducible hernia, 110 (24.3%) had irreducible hernia, 84 (18.6%) and 50(11.1%) patients had obstructed and strangulated hernias respectively. The majority of patients (53.1%) had right sided inguinal hernia with a right-to-left ratio of 2.1: 1. Ninety-two (20.4%) patients had bilateral inguinal hernias. 296 (65.5%) patients had indirect hernia, 102 (22.6%) had direct hernia and 54 (11.9%) had both indirect and direct types (pantaloon hernia). All patients in this study underwent open herniorrhaphy. The majority of patients (61.5%) underwent elective herniorrhaphy under spinal anaesthesia (69.2%). Local anaesthesia was used in only 1.1% of cases. Bowel resection was required in 15.9% of patients. Modified Bassini’s repair (79.9%) was the most common technique of posterior wall repair of the inguinal canal. Lichtenstein mesh repair was used in only one (0.2%) patient. Complication rate was 12.4% and it was significantly higher in emergency herniorrhaphy than in elective herniorrhaphy (P = 0.002). The median length of hospital stay was 8 days and it was significantly longer in patients with advanced age, delayed admission, concomitant medical illness, high ASA class, the need for bowel resection and in those with surgical repair performed under general anesthesia (P < 0.001). Mortality rate was 9.7%. Longer duration of symptoms, late hospitalization, coexisting disease, high ASA class, delayed operation, the need for bowel resection and presence of complications were found to be predictors of mortality (P < 0.001).

**Conclusion:**

Inguinal hernias continue to be a source of morbidity and mortality in our centre. Early presentation and elective repair of inguinal hernias is pivotal in order to eliminate the morbidity and mortality associated with this very common problem.

## Background

Inguinal hernia is among the most common problems encountered by general surgeons and may have significant complications [1]. Globally, inguinal hernia is the most common type of hernia, comprising of approximately 75% of all abdominal wall hernias [1-3]. Inguinal hernia repair is one of the most common general surgical operations worldwide accounting for 10 to 15% of all surgical procedures and is the second most common surgical procedure after appendicectomy [3,4]. It has been estimated that worldwide over 20 million repairs of inguinal hernia are carried out each year, the specific operation rates varying between countries from around 100–300 per 100 000 population per year [5]. In the United Kingdom some 100 000 inguinal hernias are repaired each year and approximately 750 000 inguinal hernias are repaired each year in the United States [6]. In parts of Africa, the annual incidence of inguinal hernia is as high as 175 per 100,000 people [7]. However, less than 40% are actually repaired, resulting in many patients developing long-standing inguinoscrotal hernias associated with a higher incidence of morbidity and mortality. In Bugando Medical Centre in Mwanza, Tanzania, inguinal hernia is the single commonest indication for admission to the surgical wards.

Inguinal hernia has a life time prevalence of 27% in men and 3% in women, which appears to drop after 45 years of age [3,8]. Male and female ratio is greater than 10:1 Two third of hernias are indirect and nearly two third of recurrent hernias are direct [8]. Inguinal hernia has approximately 10% incidence of incarceration and a portion of these may be strangulated. Recurrence rate is less than 1% in children and vary in adult according to the method of repair [8,9].

Since Bassini published his original description of inguinal hernia repair in 1887, many techniques for hernia repair such as Shouldice, Darning, Desarda, Modified Bassini, Lichtenstein mesh repair and the more recent laparoscopic repair have been published [4,5,8-11]. Laparoscopic and Lichtenstein mesh repair are becoming popular in recent days [11] as they are associated with rapid return to normal activities with low recurrence rates [12]. However, because of poor socioeconomic status, non-affordability of patients and non-availability of mesh and laparoscopy in most centres in developing countries, these inguinal hernial repair techniques are not popular in these countries as a result traditional suturing techniques such as Shouldice, Darning, Desarda and Modified Bassini are still practiced widely in this part of the world [13,14].

The management of inguinal poses therapeutic challenges to general surgeons practicing in resource-limited countries [15]. Late presentation of the disease coupled with lack of modern therapeutic facilities such as laparoscopy and mesh are among the hallmarks of the disease in developing countries [7,15]. Indeed, in many parts of Africa many patients develop large inguinoscrotal herniation as a result of delayed presentation, and the need for emergency surgery with its attendant mortality is not uncommon. In these countries, approximately 65% of inguinal hernias are repaired as emergencies, with a bowel resection rate of 24% and mortality of 87% in those not reaching hospital [14-16]. Early presentation and elective repair of inguinal hernia have been reported to eliminate the morbidity and mortality associated with this very common problem [7,16].

There is paucity of published data on surgical management of inguinal hernias in our environment as there is no local study which has been done in any hospital in Tanzania and Bugando Medical Centre in particular. This study was undertaken to describe our own experiences in the surgical management of inguinal hernias in our local environment, outlining the clinical profile, treatment outcome and identify predictors of outcome among these patients.

## Methods

### Study design and setting

This was a descriptive prospective study of patients who were operated for inguinal hernias at Bugando Medical Centre (BMC) between April 2010 and March 2012. BMC is located in Mwanza city along the shore of Lake Victoria in the northwestern part of Tanzania. It is a tertiary care and teaching hospital for the Catholic University of Health and Allied Sciences-Bugando (CUHAS-Bugando) and has 1000 beds. BMC is one of the four largest referral hospitals in the country and serves as a referral centre for tertiary specialist care for a catchment population of approximately 13 million people from Mwanza, Mara, Kagera, Shinyanga, Tabora and Kigoma.

### Study subjects/population

All patients who presented to the surgical wards and clinics with a clinical diagnosis of inguinal hernias and subsequently underwent surgery were included in the study. Patients presenting with inguinal hernias associated with obstructive uropathy, chronic obstructive airway disease or chronic constipation and those who refused to give consent were excluded from the study.

Recruitment of patients to participate in the study was done at the Accident & Emergency (A&E) department and in surgical wards and clinics. The clinical diagnosis of inguinal hernia was made by detailed history and clinical examination. Patients who were scheduled to undergo emergency surgery were admitted through A&E department after thorough resuscitation and patients scheduled for elective surgery were admitted a day before surgery through surgical outpatients.

Pre-operatively, all patients were assessed for fitness for surgery and anaesthesia. Each patient was classified according to the physical status scale of the American Society of Anesthesiologists or ASA class [17], which assigns a risk level for surgery and anesthesia. Those regarded as unfit for general anaesthesia were labeled as candidates for the procedure under local anaesthesia. A day before surgery the patients were subjected to clear fluid diet and were advised nil per oral regimen the midnight before surgery day. Surgery was performed under local, spinal, or general anesthesia in accordance with the patient’s physiological status and the anesthetist’s opinion.

Following incision the external oblique aponeurosis was cut exposing the inguinal canal and its contents. Then dissection of sac from the spermatic cord and herniotomy was performed leaving entire floor and posterior wall of the inguinal canal exposed for repair. The method of posterior wall repair was determined by the individual surgeon’s preference. All wounds, except those closed subcutaneously, were closed with non-absorbable (nylon) sutures. Operative findings were noted and postoperative course and findings were noted. Postoperatively all patients were given a full dose of parenteral narcotic analgesic such as Pethidine. Antibiotics commonly ampiclox and gentamicin were also prescribed in all patients. Daycare patients were discharged home on the day of operation when it was ascertained that they had fully recovered from anaesthesia. Inpatients were discharged two to five days after surgery depending on the physiological status of the patients. All patients were reviewed on the seventh day after operation. During this first visit, the wounds were inspected and stitches were removed. Patients were subsequently followed up for 1^st^ month, 3^rd^ and 6th months postoperatively for any complaints or wound complications. Data were collected using a pre-tested, coded questionnaire and included socio-demographic data (age, sex, and occupation), clinical presentation (duration of hernia, side affected, extent, reasons for late presentation, type of hernia, whether primary or recurrence, ASA status), type of surgical procedure, postoperative complications, the duration of hospital stay and mortality.

### Statistical data analysis

Statistical data analysis was done using SPSS software version 17.0 (SPSS, Inc, Chicago, IL). Data was summarized in form of proportions and frequent tables for categorical variables. Continuous variables were summarized using means, median, mode and standard deviation. P-values were computed for categorical variables using Chi-square (χ2) test and Fisher’s exact test depending on the size of the data set. Independent student t-test was used for continuous variables. Multivariate logistic regression analysis was used to determine predictor variables that are associated with outcome. A p-value of less than 0.05 was considered to constitute a statistically significant difference.

### Ethical consideration

Ethical approval to conduct the study was obtained from the CUHAS-Bugando/BMC joint institutional ethic review committee before the commencement of the study. Informed consent was sought from each patient before being enrolled into the study.

## Results

### Socio-demographic data

A total of 594 patients underwent abdominal hernial repair during the study period. Of these, 452 (76.1%) were due to inguinal hernia, representing 13.3% of all the general surgical operations (i.e. 5966) done during the period under review. Their ages ranged from 3 months to 78 years with a median age of 36 years. 440 (97.3%) were males and 12 (2.7%) were females with a male to female ratio of 36.7:1. This gender deference was statistically significant (P = 0.003). The majority of patients, 341 (75.4%) were peasants coming from the rural areas located a considerable distance from Mwanza City and most of them, 372 (82.3%) had either primary or no formal education. Major pre-morbid medical illness was found in 76 (16.8%) patients. Of these, 24 (31.6%) had chronic chest infections, 13 (17.1%) had hypertension, 12 (15.8%) had diabetes mellitus, 10 (13.2%) patients had urinary tract infections, 7 (9.2%) patients had sickle cell disease, 6 (7.9%) patients had portal hypertension with ascites and 4 (5.3%) patients had cardiovascular disorders. All these concomitant medical illness were controlled before operation. A history of having lifted heavy weights was ascertained in117 (25.9%) patients. Sixty nine (15.3%) patients were heavy smokers and 17 (37.6%) patients were treated for obstructive uropathy (i.e. bouginage, DVU, prostatectomy).

### Clinical presentation

The duration of hernia ranged from 1 day to 24 years with a median duration of 6 years and the majority of patients, 202(44.7%) presented more than five years of onset of hernia. One hundred and ten (24.3%) patients presented less than one year of onset of illness and 140 (31.0%) patients presented between one and five years of onset of illness. The reasons for late presentation are shown in Table 1.

**Table 1:** The reasons for late presentation (N=312)

**Reasons for late presentation**	**Frequency**	**Percentage**
Financial constraints	298	95.5
Lack of awareness of the disease	267	85.6
Long distance from health facilities	121	38.8
Fear of surgery	95	30.4
Feeling that hernia is not a dangerous disease	24	7.7
Treated by traditional healers	12	3.8
No reason reported	122	39.1

Three hundred and eighteen (70.4%) patients presented with primary hernia and 134 (29.6%) patients had recurrences from previous open operations. Inguinoscrotal hernia was the commonest; occurring in 302 (66.8%) patients. 150 (33.2%) patients had inguinal hernia. At presentation, 208 (46.0%) patients had reducible hernia, 110 (24.3%) had irreducible hernia, 84 (18.6%) and 50(11.1%) patients had obstructed and strangulated hernias respectively. Four (0.9%) patients who had strangulated Richter’s hernia presented with enterocutaneous fistula from perforation.

Two hundred and forty (53.1%) patients had right sided inguinal hernias and 114 (25.2%) had left sided hernias with a right-to-left ratio of 2.1: 1. Ninety-two (20.4%) patients had bilateral inguinal hernias. 296 (65.5%) patients had indirect hernia, 102 (22.6%) had direct hernia and 54 (11.9%) had both indirect and direct types (pantaloon hernia).

### Admission pattern, anaesthetic assessment and surgical treatment

The majority of patients, 278 (61.5%) were admitted through surgical outpatient clinic and the remaining 174 (38.5%) patients were admitted through the Accident & Emergency department. Forty-two (9.3%) patients were, after operation, admitted in the Intensive Care Unit (ICU) before being admitted to the general surgical wards. Out of these, 28 (66.7%) were subjected to ventilatory support for a median duration of 7 days (range 1–16 days). According to ASA classification, 284 (62.8%) patients had ASA class I, 81 (17.9%) had ASA class II, 70 (15.5%) had ASA class III, 12 (2.7%) had ASA class IV and 5 (1.1%) patients had ASA class V. A high ASA score was found to be an independent predictor of gangrenous bowel (P= 0.000).

All patients in this study underwent open herniotomy/herniorrhaphy. Laparoscopic herniorrhaphy was not performed in our patients due to lack of facilities for laparoscopic surgery at our centre. Elective herniorrhaphy was performed in 278 (61.5%) patients and the remaining 174 (38.5%) patients had emergency herniorrhaphy. Herniorrhaphy was performed under spinal anesthesia in 313 (69.2%) patients, general anesthesia in 134 (29.4%) patients and local anesthesia in five (1.1%). A shift from spinal anesthesia to general anaesthesia was recorded in 34 (7.5%) patients. Five (1.1%) individuals were managed on day case basis.

Operative findings were mainly indirect hernia sacs in 296 (65.5%) patients and direct sacs in 102 (22.6%) patients. Fifty-four (11.9%) patients had both indirect and direct hernial sacs. The contents of the hernial sac are shown in Table 2. Gangrenous bowel was found in 68 (15.0%) patients. The types of indirect inguinal hernia were classified intra-operatively as follows: bobunocoele in 70 (15.5%) patients, funicular in 80 (17.7%) and inguino-scrotal (complete) in 302 (66.8%) patients. Bowel resection was required in 72 (15.9%) patients (i.e. 68 patients due to gangrenous bowel and 4 patients due to multiple perforations).

**Table 2:** The contents of the hernial sac (N= 452)

**Contents of the hernial sac**	**Frequency**	**Percentage**
Loops of small bowel	112	24.8
Omentum	92	20.4
Large bowel (colon)	21	4.6
Sliding hernia	4	0.9
Appendix	3	0.7
Urinary bladder	2	0.4
Richter’s hernia	2	0.4
None	142	31.4

The repair posterior wall of inguinal canal was employed by modified Bassini’s repair in 361(79.9%) patients, Shouldice repair in 4(0.8%), Darning and Desarda repair in 3 (0.7%) patients each respectively. Only one (0.2%) Indian patient had Lichtenstein mesh repair. Herniotomy only was done in 79 (37.7%) paediatric patients. The overall median operative duration was 72 minutes (range 34 to 150 minutes).The median duration of surgery in patients with unilateral hernias was 48 minutes (range = 28–95 minutes) while the median duration of surgery in those with bilateral hernias was 62 minutes (range 40–98 minutes). This difference was not statistically significant (*P* =0.566).

### Outcome of surgical treatment

In this study, a total of 62 post-operative complications were recorded in 56 (12.4%) patients. Of these, surgical site infection was the most common post-operative complication accounting for 38.7% of cases (Table 3). Complication rate was significantly higher in emergency herniorrhaphy than in elective herniorrhaphy (34.6% versus 15.9%) (P = 0.002). All complications resolved on conservative treatment alone except in four patients who required re-operation for intestinal obstruction and peritonitis respectively.

**Table 3:** Post-operative complications (N= 62)

**Post-operative complications**	**Frequency**	**Percentage**
Surgical site infection	24	38.7
Scrotal hematoma	16	25.8
Postoperative pyrexia	10	16.1
Paralytic ileus	7	11.3
Intestinal obstruction requiring re-operation	2	3.2
Peritonitis requiring re-operation	2	3.2
Enterocutaneous fistula	1	1.6
**Total**	**62**	**100**

The length of hospital stay (LOS) ranged from 4 to 34 days (median 8 days).The LOS for non-survivors ranged from 1 day to 11 days (median 4 days). The length of ICU stay ranged from 1 to 13 days (median 5 days). The length of hospital stay was significantly longer in patients with advanced age, delayed admission, concomitant medical illness, high ASA class, the need for bowel resection and in those with surgical repair performed under general anesthesia (P < 0.001).

In this study, forty-four patients died giving a mortality rate 9.7%. Longer duration of symptoms, late hospitalization, coexisting disease, high ASA class, delayed operation, the need for bowel resection and presence of complications were found to be predictors of mortality (P < 0.001).

Out of 408 survivors, 269 (65.9%) patients, 102 (25.0%) and 37 (9.1%) patients were available for follow up at 3 months, 6 months and 1 year respectively. The median duration of post-operative follow up was eight months (1–24 months). Chronic groin pain was recorded in 12 (2.8%) post-repairs at the surgical out patient clinic. All improved on mild analgesics. There were only three (0.7%) cases of hernia recurrences (one post herniotomy and two post-Bassini repair).

## Discussion

Inguinal hernia repair is a commonly performed general surgical operation, and therefore comprises a significant proportion of surgical workload in many centres [1,3,18-20]. Inguinal hernias are undoubtedly the commonest hernia type. Our results showed that inguinal hernia comprised of 76.1% of all abdominal wall hernias, a figure slightly higher than the 75% quoted by various authors [1-3]. Inguinal hernia repair has been reported to constitute approximately 10-15% of all surgical procedures performed in a general surgical unit [3,4]. In our study, inguinal hernia repair constituted 13.3% of all the adult general surgical operations which slightly higher than 12.5% reported by Garba [1] in Nigeria.

In this study, the youngest patient was only 3 months old. This confirms the congenital origin of some of these hernias, many of which are never brought to the attention of a clinician until they become symptomatic. The median age of 36 years was similar to that described in a previous report [21], but at variant with other studies which reported high age incidence [19,22]. We were unable to find any reason for this age difference. High incidence of the disease in this age has a negative impact on the country’s economy because this group represents the economically productive age group and portrays an economic lost both to the family and the nation.

Inguinal hernias are quoted as being more than 20 times more common in males than females [2]. Our results are similar, in fact showing that inguinal hernia repairs were carried out in total almost 37 times more commonly in males than females. The exact reason for this male preponderance is not known although it is possible that males have an increased risk of hernial development due to their involvement in strenuous work which is responsible for development of hernias.

Inguinal hernia has been reported to be more prevalent in people with low socio-economic status [21]. This observation is reflected in our study where most of patients had either primary or no formal education and the majority of them were peasants coming from the rural areas located a considerable distance from Mwanza City. Similar observation was reported by others [1,21,23]. This observation has an implication on accessibility to health care facilities and awareness of the disease.

Concomitant diseases in patients with inguinal hernias have been reported to be associated with poor outcome [21,23,24]. In the present study, concomitant medical illness was an important determinant of morbidity. Moreover, this factor reached almost the statistical significance for mortality. The length of hospital stay was also encountered to be longer in patients with concomitant diseases.

The clinical presentation of inguinal hernia in our patients is not different from those in other developing countries [1,15,21,24,25]. In many developing countries, lack of awareness and financial constraints make many patients present very late with giant inguinoscrotal hernia which is a serious life-threatening condition [21]. A long standing history of inguinal hernia is common and presentation during bubonocele and funicular stages are rare [26,27]. This finding is reflected in our study where most of patients presented late with complete inguinoscrotal hernia and some presented for the first time with obstructed or strangulated hernias. As in other studies done in other developing countries [21,24,25], financial constraints and lack of awareness were reported as the most common reasons for the late presentation in our study.

The finding that inguinal hernias were more preponderant in the right side is in agreement with similar observations in earlier series [1,25]. The ratio of right inguinal hernia to left inguinal hernia in our study was 2.1: 1. The later descent of the right testis and a higher incidence of failure of closure of the processus vaginalis are factors usually ascribed to the predilection of this disease on the right [1,2,25].

In agreement with other studies done in developing countries [1,21,25], all patients in this study were managed by open herniorrhaphy of which Modified Bassini’s repair was the method of choice in majority of cases operated except in most paediatric hernias for which Herniotomy was done. Simplicity, speed of execution and the preference of the surgeon probably accounted for the popularity of Bassini approach in this centre. Since Lichtenstein presented his open mesh repair technique for inguinal hernia in 1986 [28], the technique has become the most commonly used on account of its ease of operation and because it provides a tension-free repair with good long-term results [8,29]. Because of high cost and its non-availability, the use of mesh in the repair of inguinal hernia is not popular in our centre, and in the present study it was used in only one patient. In resource-poor African sub-region, the cost of mesh, not to mention its acquisition, might represent a formidable barrier to its use [8,9,14].

Laparoscopic repair was not performed in this study similar to what reported in other centres in most developing countries [1,21,25]. Laparoscopic repair has become a popular method for treating inguinal hernias in developed countries owing to its superior benefits relating to reduced morbidity, shorter hospital stay, accelerated recovery and earlier return to previous employment [30,31]. This form of treatment may become feasible in our health community as soon as the facilities and requisite expertise are made available.

The choice of anaesthesia in hernia surgery is determined by the physiological state of the patient, size of the hernia and extent of the perceived procedure. In the present study, spinal anaesthesia was the single most common aesthesia used which is in contrast to McFarlane [32] who advocated use of local anaesthesia in patients with small uncomplicated inguinal hernia. The use of local anaesthesia was not feasible in our study because majority of patients presented with huge inguinal scrotal hernias which required either spinal or general aesthesia for full muscle relaxation. The choice of anaesthesia also influences the practice of day case hernia surgery. Day case herniorrhaphy has been found to be feasible, safe, effective and with the befit of earlier ambulation when dealing with the uncomplicated average sized hernia in a physiologically fit individual [25,31,33]. The day case hernia surgery in this study was practiced in only 1.1% of cases as majority of our patients presented late with huge inguinoscrotal hernia which needed local or general anaesthesia and at least a day or two days of hospitalization postoperatively. Success of day case hernia surgery relies on careful patient selection, skillful operative techniques, safe anaesthesia, and adequate postoperative care. Continuing audit is essential to maintain and improve the quality and standard of ambulatory surgery provided.

In the present study, bowel resection rate in patients with obstructed or strangulated hernias was 15.9%. The need for bowel resection closely correlated with the time interval between the onset of acute symptoms and subsequent operation.

In this study, the complication rate of 12.4% is slightly higher than the finding of between 4.2% and 12% in other series [25,34,35]. Complication rate was significantly higher in emergency herniorrhaphy than in elective herniorrhaphy. High complication rate in emergency herniorrhaphy may be explained by the fact that the majority of patients in the present study presented late with various late complications of hernia which required emergency surgical treatment. Also, the fact that most emergency herniorrhaphy in the present series were performed by basic surgical trainees who were the first on call may explain the high rate of complications in this group of patients. In order to reduce the incidence of complications that follow emergency herniorrhaphy, adequate supervision of surgical trainees must be enforced as well as strict attention to aseptic technique and meticulous haemostasis.

The overall median duration of hospital stay in the present study was 9 days which is slightly higher than that reported by Mbah [25] in Nigeria. The predictors of the length of hospital stay in this study were advanced age, delayed admission, concomitant medical illness, high ASA class, the need for bowel resection and in those with surgical repair performed under general anesthesia. Prolonged duration of hospital stay has an impact on hospital resources as well as on increased cost of health care, loss of productivity and reduced quality of life.

The overall mortality rates from inguinal hernia have been reported in literature to range from 1- 14% [1,35]. The mortality rate of 9.7% in the present study is higher than the rate of 5.3% reported by Mbah [24] and that of 3.4% reported by Alvarez *et al.*[36]. In this study, longer duration of symptoms, late hospitalization, coexisting disease, high ASA class, delayed operation, the need for bowel resection and presence of complications were found to be significant factors associated with high mortality.

In the present study, the incidence of chronic groin pain during follow up period was low in comparison with rates varying from 0% to 54% reported in Western literature after laparoscopic and open mesh hernioplasty [20,37]. This complication of mesh repair appears to be much lower after Bassini herniorrhaphy probably for technical reasons.

Short period of follow up was the potential limitation of this study as it was not possible to assess the long term outcome of various modalities of surgical treatments.

## Conclusion

Inguinal hernias continue to be a source of morbidity and mortality in our centre. Late presentation of the disease and lack of modern therapeutic facilities such as laparoscopy and mesh are among the hallmarks of the disease in this part of Tanzania. Modified Bassini’s repair was the method of choice in majority of cases operated. It is therefore recommended that early presentation and elective repair of inguinal hernia is of paramount importance in order to eliminate the morbidity and mortality associated with this very common problem.

## Competing interests

Both declare that they have no competing interests.

## Authors’ contributions

JBM conceived the study and did the literature search, coordinated the write-up, participated in data analysis and editing. PLC participated in writing the manuscript, data analysis, editing and submission of the article. Both authors read and approved the final manuscript.
